# Quiet standing: The Single Inverted Pendulum model is not so bad after all

**DOI:** 10.1371/journal.pone.0213870

**Published:** 2019-03-21

**Authors:** Pietro Morasso, Amel Cherif, Jacopo Zenzeri

**Affiliations:** 1 Department of Robotics, Brain and Cognitive Sciences, Istituto Italiano di Tecnologia, Genoa, Italy; 2 Department of Informatics, Bioengineering, Robotics, and System Engineering, University of Genoa, Genoa, Italy; Fondazione Santa Lucia Istituto di Ricovero e Cura a Carattere Scientifico, ITALY

## Abstract

In the study of balance and postural control the (Single) Inverted Pendulum model (SIP) has been taken for a long time as an acceptable paradigm, with the implicit assumption that only ankle rotations are relevant for describing and explaining sway movements. However, more recent kinematic analysis of quiet standing revealed that hip motion cannot be neglected at all and that ankle-hip oscillatory patterns are characterized by complex in-phase and anti-phase interactions, suggesting that the SIP model should be substituted by a DIP (Double Inverted Pendulum) model. It was also suggested that DIP control could be characterized as a kind of optimal bi-axial active controller whose goal is minimizing the acceleration of the global CoM (Center of Mass). We propose here an alternative where active feedback control is applied in an intermittent manner only to the ankle joint, whereas the hip joint is stabilized by a passive stiffness mechanism. The active control impulses are delivered to the ankle joint as a function of the delayed state vector (tilt rotation angle + tilt rotational speed) of a Virtual Inverted Pendulum (VIP), namely a pendulum that links the ankle to the CoM, embedded in the real DIP. Simulations of such DIP/VIP model, with the hybrid control mechanism, show that it can reproduce the in-phase/anti-phase interaction patterns of the two joints described by several experimental studies. Moreover, the simulations demonstrate that the DIP/VIP model can also reproduce the measured minimization of the CoM acceleration, as an indirect biomechanical consequence of the dynamic interaction between the active control of the ankle joint and the passive control of the hip joint. We suggest that although the SIP model is literally false, because it ignores the ankle-hip coordination, it is functionally correct and practically acceptable for experimental studies that focus on the postural oscillations of the CoM.

## Introduction

Bipedal upright standing on a stable surface is an ability acquired early in life and performed in a fully automatic manner without any degree of attentional effort. Apparently, it seems hardly a worth topic in the study of balance and postural control, except for the clinical setting, given the simplicity of an experimental investigation typically based on force platform measurement of involuntary sway movements in the sagittal plane. The implicit assumption underlying the clinical interest of body sway is that the projection of the body center-of-mass on the standing surface (CoM) is the regulated variable of the postural control system, which can be indirectly accessed by measuring the position of the center-of-pressure (CoP). A number of performance measures of maintenance of this posture, based on the CoP-CoM pair, have been developed which are used in clinical decision making [1] in relation with a number of pathological conditions, such as the following ones: cerebellar ataxia [2–4], vestibular dysfunctions [5–7], peripheral neuropathy due to diabetes [8–10], Alzheimer's disease [11,12], multiple sclerosis [13,14], Parkinson’s disease [15,16], traumatic brain injury [17,18], stroke [19,20] and identification of malingerers for forensic medicine [21].

The underlying biomechanical model is a Single Inverted Pendulum (SIP), pivoted around the ankle. In this framework, sway movements are interpreted as back and forth oscillations of the SIP under the action of opposing forces, namely the destabilizing force of gravity, counteracted by the stabilizing effect of ankle muscles. However, the mechanisms and control principles involved were and are not evident and are still topics of scientific dispute. In this framework, an influential proposal is the muscle stiffness control model by Winter et al. [22] which may be considered as a member of the family of control models related to the Equilibrium Point Hypothesis (EPH) [23–25]. The attractive feature of Winter’s model, common to all the members of the EPH family, is that it offers a simple control scheme for regulation of posture: it exploits the mechanical properties of muscles by providing almost instantaneous corrective response to disturbances, thus reducing the operating demands on the Central Nervous System (CNS). The crucial element of the model, in the case of upright standing, is the stiffness value of the ankle joint, in comparison with the rate of growth of the toppling torque due to gravity which thus identifies a critical value of stiffness. As a matter of fact, different direct methods for evaluating the ankle stiffness [26,27] demonstrated that ankle stiffness is clearly under-critical. A similar conclusion was reached by van Soest et al. [28] on the basis of a detailed neuromuscular model: they found that even at maximal co-contraction levels of the ankle muscles the joint stiffness is insufficient to achieve a locally stable system. This result was further supported by specific measurements of the stiffness of the Achilles tendon [29] that exhibited a compliance level incompatible with the stiffness control hypothesis, taking into account that this important tendon is serially connected to the ankle plantar flexor muscles. It is also worth mentioning that the inefficiency of attempting to modulate joint stiffness via co-contraction of antagonist muscles is peculiar of the ankle joint, thus highlighting the specificity of the control mechanisms of the bipedal standing posture.

As a consequence of the insufficient physiological level of the ankle stiffness, it became clear that it was necessary to supplement such passive compensation mechanism of gravity-driven instability with suitable active control strategies. Many approaches have been investigated for solving this problem and the most simple solution adopted by a number of researchers was a conventional, linear, continuous-time feedback controller, based on proportional and derivative feedback (continuous *PD* control model) [30–35]. The cybernetic problem here is that such feedback information is delivered to the spinal and supra-spinal control centers through multiple sensory channels (proprioceptive, cerebellar, and visual) with a significant delay, well exceeding 0.2 s. In such conditions the *PD* control parameters must be tuned carefully by taking into account two contrasting constraints: 1) the constraint of static stability, that dictates a minimum value of the *P* parameter as a function of the gravity toppling influence, and 2) the constraint of dynamic stability, that imposes an upper bound for the *PD* parameters, stronger and stronger as the delay increases.

In order to improve the robustness of the *PD* control paradigm, an intermittent version was proposed [36–38], characterized by a simple switching mechanism defined in the phase plane of the SIP ($q\;\mathrm{vs}.\dot{q}$, where *q* is the ankle rotation angle). As a matter of fact, there is ample evidence suggesting the discontinuous nature of the feedback control action in upright standing. Consider, for example, the analysis of posturographic patterns [39–41], EMG signals [26,38,42], and the non-uniform character of sway path [43]. From the computational point of view, the power of the intermittent control strategy for stabilizing the SIP system is that it exploits an implicit “affordance”, provided by the intrinsic dynamics of such tasks, namely the fact that the uncontrolled SIP is characterized by a saddle-like instability, including a stable and unstable manifold in the phase plane: when the driving action is switched off, the state vector is attracted to the equilibrium configuration, if the vector is closer to the stable than to the unstable manifold, whereas it is repulsed away in the opposite case. This “affordance” suggested to adopt an alternation strategy between an off-phase, in the former case, and an on-phase, in the latter case, as reported in previous studies [36–38]. Remarkably, this strategy can succeed to achieve bounded stability, driving the sway patterns towards a limit cycle, even if the dynamics of the on-phase is unstable when applied continuously, thus increasing in a substantial way the size of the stability area in the space of control parameters in comparison with a conventional continuous control paradigm [37,38]. Moreover, it is worth noting a crucial difference between the continuous and intermittent paradigms: in the former case, the target of the controller is the unstable upright condition, whereas, in the latter case, the target is the whole stable manifold, with the simple decision paradigm to switch off the control action if the state vector is sufficiently close to it.

On the other hand, the SIP model has been challenged by several recent studies clearly showing that movement around the hip joint are not negligible [35,44,45]: in particular, the range of variation of the angular displacement, velocity, and acceleration of the hip were demonstrated to be significantly greater than those of the ankle, with a systematic increase of the ankle-hip difference from angular variations to the corresponding first and second derivative. As a consequence, it has been suggested that the SIP model should be substituted by a multi-link paradigm, at least a Double Inverted Pendulum (DIP) model, involving the coordinated control of ankle and hip joints. Ankle-hip joint coordination patterns have been analyzed both in the time and frequency domain. In the former case it was found that the acceleration profiles are strongly characterized by anti-phase patterns; the same holds, to a smaller degree, also for the velocity profiles, whereas the rotational profiles exhibit an overall mild in-phase correlation [44]. Moreover, in the frequency domain, the rotational profiles of the two joints appear to be characterized by co-existing coordination patterns (in-phase and anti-phase, respectively) after a suitable frequency analysis [35,45]: the leg and trunk segments of the body move in-phase at low frequency (below 0.5 Hz) but they switch to anti-phase coordination at high frequency (above approximately 0.9 Hz).

Having accepted the fact that the SIP model misses part of the observable behavior, it remains an open question the origin of the coordination between the two body segments of the DIP model, in terms of fundamental mechanisms and control principles involved. We believe that this question can be met in an efficient manner by extending the SIP model rather than rejecting it. The idea is to associate a single degree of freedom Virtual Inverted Pendulum (VIP) to the actual DIP. The VIP is an inverted pendulum that connects the ankle joint to the overall CoM of the body: the oscillations of this virtual pendulum, namely the rotations of the virtual ankle, are functions of the real rotation patterns of the two joints of the DIP model, i.e. ankle and hip. Although this inverted pendulum does not exist physically, its oscillations are directly perceivable by standing subjects through the CoP, namely by means of a combination of tactile and proprioceptive sensors of the feet. In this manner, it is possible to regulate the stabilization of the ankle joint with a control mechanism which is quite similar to the one already studied for the SIP model, namely an intermittent feedback controller complementing the intrinsic stiffness of the ankle muscles. As regards the hip joint we suggest a stiffness strategy similar to the one originally proposed by Winter et al. [46]. This strategy for the hip joint is feasible because there is not the limitation of the Achilles tendon in the ankle joint; moreover, the critical stiffness value is strongly smaller for the hip than for the ankle case for purely biomechanical reasons, thus requiring a very small amount of co-contraction of the hip muscles for achieving a working level of hip stiffness. Specifically, for an inverted pendulum the gravity-driven toppling torque is τ_g_ = mgh sin(q) ≈ mgh q and thus the critical value of stiffness is K_crit_ = mgh where m is the mass of the pendulum and h is the distance between the hinge of the pendulum and the CoM. Thus, the critical stiffness of the whole body hinged around the ankle is much greater than the critical stiffness of the upper body, hinged around the hip. The biological plausibility of this strategy is also consistent with the coherence analysis of muscle activity during quiet stance [47] which shows a lack of correlation between the oscillations of the trunk and the activity of the muscles, which exert direct control over it.

Summing up, we propose a hybrid control of the VIP/DIP model: intermittent active control of the ankle (via the VIP part of the model) and passive stiffness control of the hip (via the DIP part). The simulations demonstrate that the proposed model is compatible with the complex inter-joint coordination patterns summarized above, without any need of explicit high-level coordination mechanisms. Moreover, SIP and VIP sway patterns appear to be quite similar and thus we are confident to claim that in spite of the fact that hip rotation is far from being negligible and indeed has larger amplitude than ankle oscillations, its role is marginal, as regards the active stabilization of upright posture around the ankle, which is the main source of instability and thus the main target of active CNS control. In this sense, we suggest that the core of the fundamental control mechanism of the upright posture is still captured by a variation of the SIP.

## Materials and methods

### DIP/VIP model

**Fig 1:**
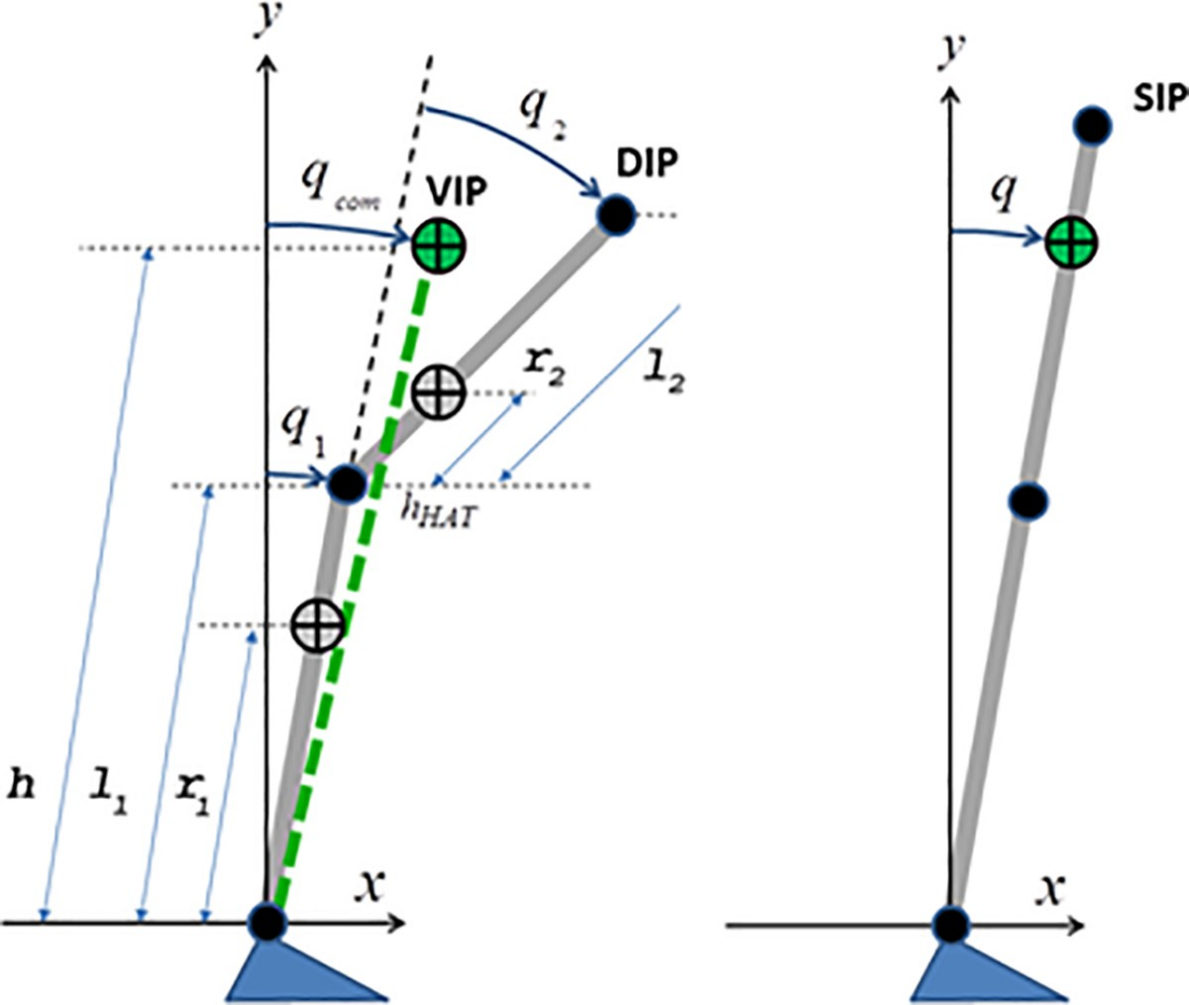
Biomechanical models. The DIP/VIP model (left panel) and the corresponding SIP model (right panel).

Fig 1 illustrates the DIP/VIP model that is used in the simulations, comparing it with the corresponding SIP model.

The DIP model has two links that are related, respectively, to the legs (total mass *m*_1_, leg-length *l*_1_, distance of the barycenter from the ankle axis *r*_1_, and moment of inertia around the barycenter *I*_1_) and to the upper body (HAT: Head-Arm-Trunk) characterized by the corresponding parameters (*m*_2_, *l*_2_, *r*_2_, *I*_2_). The two degrees of freedom are the ankle rotation angle (*q*_1_) and the hip angle (*q*_2_).

The VIP model has a single degree of freedom (*q*_*com*_) and consists of a single virtual inverted pendulum that links the ankle to the global CoM of the DIP model; its mass equals the total mass of the DIP (*m* = *m*_1_ + *m*_2_) and *h* denotes the corresponding length.

The DIP model fully characterizes the biomechanics of the standing body and the associated VIP model is instrumental for active control based on the intermittent paradigm. The dynamic equations of the DIP model can be obtained by using the Lagrangian approach that yields the following non-linear ODE:

$$M(q)\ddot{q}+C(q,\dot{q})\dot{q}+G(q)=\tau$$ (1)

For simplicity we use the following synthetic notation: *s*_1_ = sin *q*_1_, *s*_2_ = sin *q*_2_, *s*_12_ = sin(*q*_1_+*q*_2_), *c*_12_ = cos(*q*_1_+*q*_2_).

***M***(*q*) is the inertia matrix that varies as a function of the hip rotation angle according to the following formula:

$$\begin{array}{l} \left\{ \begin{array}{l} M_{11}=a+2b\;c_{2}\\ M_{12}=M_{21}=d+b\;c_{2}\\ M_{22}=d \end{array}\right.\\ \left\{ \begin{array}{l} a=I_{1}+I_{2}+m_{1}{r_{1}}^{2}+m_{2}({l_{1}}^{2}+{r_{2}}^{2})\\ b=m_{2}\;l_{1}\;r_{2}\\ d=I_{2}+m_{2}\;{r_{2}}^{2} \end{array}\right. \end{array}$$ (2)

$C(q,\dot{q})\dot{q}$ represents the Coriolis and centrifugal generalized forces:

$$\left\{ \begin{array}{l} C_{11}=-b\;s_{2}\;{\dot{q}}_{2}\\ C_{12}=-b\;s_{2}({\dot{q}}_{1}+{\dot{q}}_{2})\\ C_{21}=b\;s_{2}\;{\dot{q}}_{2}\\ C_{22}=0 \end{array}\right.$$ (3)

***G***(*q*) is related to the gravity dependent torques (*g* is the gravity acceleration):

$$G(q)=-g\left[\begin{array}{l} (m_{1}\;r_{1}+m_{2}\;l_{1})s_{1}+m_{2}\;r_{2}\;s_{12}\\ m_{2}\;r_{2}\;s_{12} \end{array}\right]$$ (4)

***τ*** is the total control torque that has the purpose to compensate the intrinsic instability of the upright posture and includes three contributions, determined by different control mechanisms: a bias torque ***τ***_*B*_, a stiffness torque ***τ***_*S*_, and an intermittent control torque ***τ***_*I*_:

$$\tau =\tau_{B}+\tau_{S}+\tau_{I}$$ (5)

A noise signal was added to the total control torque, with a comparable power and a frequency band limited to 10 Hz.

#### The feed-forward bias torque *τ*_*B*_

It compensates for the toppling torque due to gravity in the reference posture *q*_*ref*_ = [*q*_1*ref*_, *q*_2*ref*_] and it is applied to both joints.

$$\tau_{B}=-g\left[\begin{array}{l} \left(m_{1}\;r_{1}+m_{2}\;l_{1})\sin q_{1ref}+m_{2}\;r_{2}\sin\left(q_{1ref}+q_{2ref}\right)\right)\\ m_{2}\;r_{2}\sin\left(q_{1ref}+q_{2ref}\right)) \end{array}\right]$$ (6)

#### The stiffness torque *τ*_*S*_

It expresses the elastic properties of ankle and hip muscles and it is attributed to both joints, relative to the same reference posture:

$$\tau_{S}=-\left[\begin{array}{c} K_{a}({q_{1}-q}_{1ref})+B_{a}\;{\dot{q}}_{1}\\ K_{h}({q_{2}-q}_{2ref})+B_{h}\;{\dot{q}}_{2} \end{array}\right]$$ (7)

where *K*_*a*_ is the ankle stiffness (with the corresponding damping factor *B*_*a*_) and *K*_*h*_ is the hip stiffness (with the corresponding damping factor *B*_*h*_). The ankle stiffness is smaller than the critical value determined by the rate of growth of the gravity-dependent toppling torque of the whole body, in agreement with the measurements of the ankle stiffness [27,48]; in contrast, the hip stiffness is hypothesized to be greater than the critical value corresponding to the upper body, according to the working hypothesis of the hybrid VIP/DIP model:

$$\left\{ \begin{array}{l} K_{a}<(m_{1}+m_{2})g\;h\\ K_{h}>m_{2}\;g\;r_{2} \end{array}\right.$$ (8)

#### The intermittent feedback control torque *τ*_*I*_: Stabilization of the VIP model

This control torque is applied only to the ankle joint as a function of the VIP angle and angular velocity (*q*_*com*_, ${\dot{q}}_{com}$). The VIP angle is reconstructed on-line from the two angles of the DIP system:

$$\left\{ \begin{array}{l} x_{com}=m_{1}\;r_{1}\;s_{1}+m_{2}\left(l_{1}+r_{2}\right)s_{12}\\ y_{com}=m_{1}\;r_{1}\;c_{1}+m_{2}\left(l_{1}+r_{2}\right)c_{12} \end{array}\Rightarrow q_{com}=\tan^{-1}\left(\frac{x_{com}}{y_{com}}\right)\right.$$ (9)

The intermittent control strategy was originally conceived, as already commented in the introductory section, for stabilizing the SIP system by extending the conventional *PD* feedback controller in order to reduce the risk of instability due to the delay of the feedback signals. The basic idea was to exploit the implicit “affordance” of saddle-like instability, namely the presence of a stable and unstable manifold in the phase plane, thus suggesting the following heuristics: to switch off the feedback control action when the state vector is closer to the stable manifold than to the unstable one and to reactivate it in the opposite case. The robustness of this control paradigm is due to the fact that, even if the active control is unstable when permanently applied, the combination of actively controlled orbit segments with orbit segments driven by intrinsic dynamics may end up in bounded oscillatory patterns. It is worth emphasizing that the target of active control in the conventional continuous *PD* paradigm is the upright unstable equilibrium configuration whereas in the intermittent paradigm it is the whole stable manifold, thus extending significantly the range of values of the *PD* parameters that can support bounded stability [37].

The working hypothesis investigated in this work was to apply it to the VIP model instead of the SIP model and then verify with the DIP model simulation if such hybrid mechanism could achieve the double goal of dynamic stabilization of the standing body and reproduction of the experimentally obtained ankle-hip coordination patterns.

More specifically, having defined $\Delta q_{\delta }=(q_{com}^{ref}-q_{com}(t-\delta t))$ as the delayed angular error of the global CoM of the DIP model, with the corresponding angular speed error $\Delta {\dot{q}}_{\delta }$, the intermittent control action is defined in the phase plane of the VIP model as follows:

$$\tau_{I}=\left[\begin{array}{c} C\\ 0 \end{array}\right],\;\mathrm{with}\;\left\{ \begin{array}{l} C=P\cdot \Delta q_{\delta }+D\cdot \Delta {\dot{q}}_{\delta }\;\mathrm{if}\;\Delta q_{\delta }\cdot \left(\Delta {\dot{q}}_{\delta }-\alpha \cdot \Delta q_{\delta }\right)>0\\ C=0\;\mathrm{otherwise} \end{array}\right.$$ (10)

Here [*P*,*D*] are the proportional and derivative parameters, respectively, of the *PD* intermittent controller, which is activated in the first and third quadrant of the VIP phase plane, with an additional small slice determined by the parameter *α*. For this parameter we used the value 0.4, in agreement with the analysis in [37].

#### Parameters of the DIP/VIP model

The anthropometric parameters, which were derived from the whole-body model described in [49], are listed in Table 1:

**Table 1:** Anthropometric parameters of the DIP model.

Leg				HAT (Head-Arm-Trunk)			
*l*_1_[m]	*r*_1_[m]	*m*_1_[kg]	*I*_1_[kg m^2^]	*l*_2_[m]	*r*_2_[m]	*m*_2_[kg]	*I*_2_[kg m^2^]
0.9	0.58	28	9.21	0.88	0.32	53	5.35

The critical levels of stiffness of the two joints, namely the stiffness values that match the coefficients of the gravity toppling torques, are given by the two following expressions:

$$\left\{ \begin{array}{l} K_{a}^{crit}=\left(m_{1}+m_{2}\right)g\;h\\ K_{h}^{crit}=m_{2}\;g\;r_{2} \end{array}\right.$$ (11)

The ankle stiffness of the DIP model, that is known to be under-critical, was set to 60% of the critical level, as suggested by direct measurements of ankle stiffness [27]. For the hip stiffness we used over-critical values in order to validate the feasibility of the hypothesis about the hybrid stabilization strategy: active at the ankle (intermittent delayed feedback) and passive at the hip (via the intrinsic visco-elastic properties of hip muscles); the default value for most simulation was twice the critical hip stiffness. The corresponding damping coefficients (*B*_*a*_ and *B*_*h*_) are not critical. The chosen value for the ankle comes from empirical measurements [27]; for the hip we chose a value in order to have a damping coefficient equal to 0.7. The robustness of the hybrid stabilization strategy was also tested by varying the hip stiffness, for which direct empirical estimates are not available, in a large range. The employed values of the visco-elastic parameters are listed in Table 2.

**Table 2:** Visco-elastic parameters of the DIP model.

Ankle		Hip	
*K*_*a*_[Nm/rad]	*B*_*a*_[Nms/rad]	*K*_*h*_[Nm/rad]	*B*_*h*_[Nms/rad]
494	30	331	30

The *PD* parameters of the intermittent controller were selected heuristically after having identified the intervals of stability.

**Fig 2:**
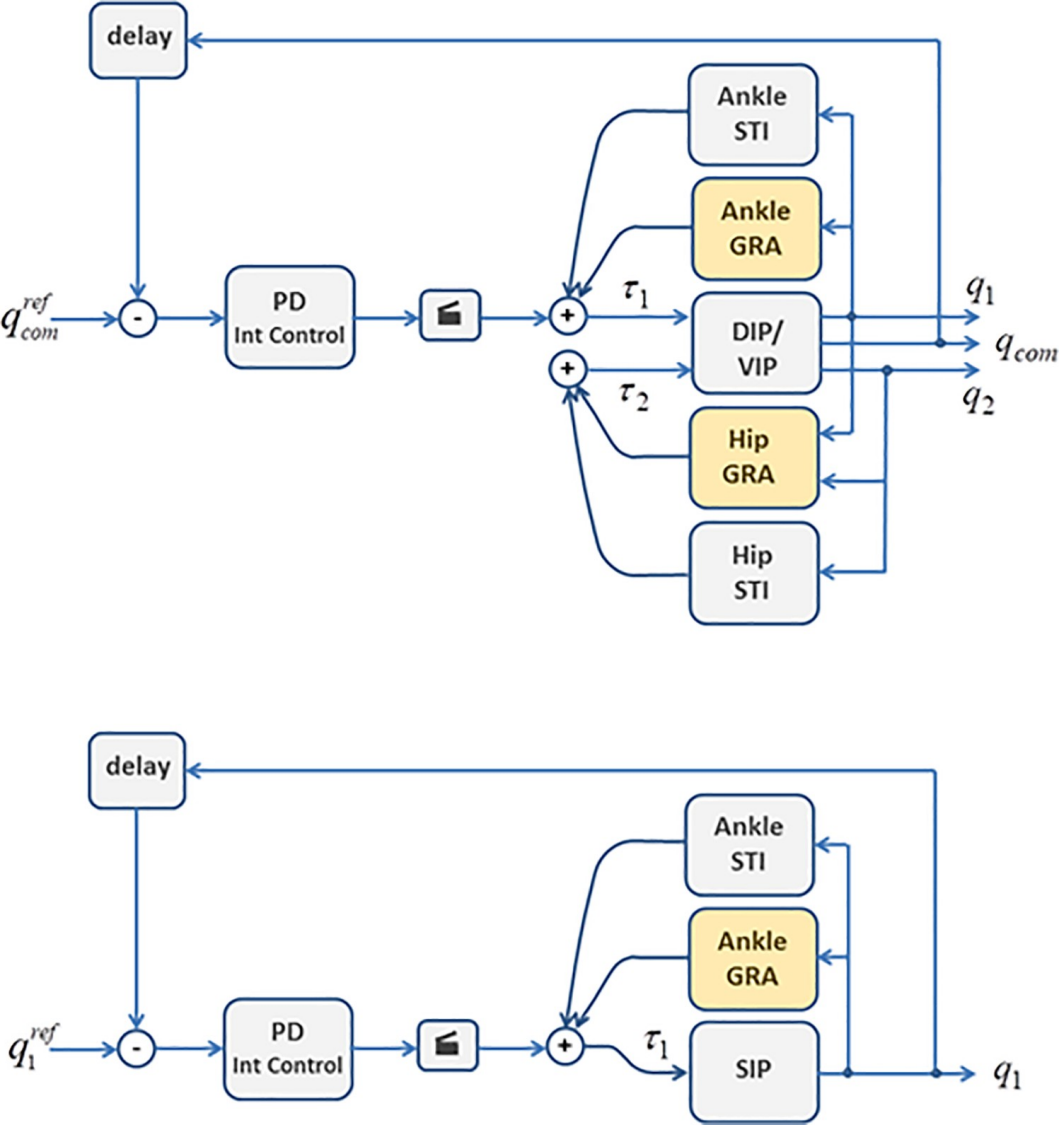
Control models. Upper panel: the hybrid DIP/VIP control model. *q*_1_: ankle rotation angle; *q*_2_: hip rotation angle; *q*_*com*_: VIP rotation angle. *τ*_1_: total ankle torque; *τ*_2_: total hip torque. GRA: Gravity torque model. STI: Stiffness torque model. The DIP/VIP block corresponds of the overall dynamics of the DIP/VIP model. Lower panel: the simplified SIP model.

According to literature on sensorimotor integration in human postural control [33,50] and cart inverted pendulum paradigm [51], the feedback delay of the intermittent *PD* controller was varied in the range of 0.20-0.25 s. In most simulations reported in the results the most challenging value (0.25 s) was used. The simulation were carried out with Matlab (MathWorks), using the forward Euler method with a time step of 0.001 s. Fig 2 (upper panel) illustrates the overall block diagram of the hybrid DIP/VIP control model.

### SIP model

Fig 1 (right panel) illustrates the SIP model. The anthropometric parameters are the same of the DIP/VIP model. Also the model equations are quite similar to the DIP/VIP model. The model has a single degree of freedom, the ankle rotation angle, whereas the hip angle is kept constant (equal to 0). The control parameters are the same, taking into account that Eq 10, namely the computation of the intermittent feedback control torque, is applied directly to the ankle joint. Fig 2 (lower panel) illustrates the block diagram of the equivalent SIP model.

## Results

In order to validate the proposed hybrid postural controller we needed to address two main issues: *robustness*, in terms of stability, and *plausibility*, in terms of inter-joint coordination. As regards the former issue we verified, by means of multiple simulations, the range of values of the *P* and *D* parameters which yielded bounded stability.

The initial condition of the simulations was characterized by a tilt angle of the ankle joint of 2 deg and a null angular velocity (plus a null angle of the hip joint for the DIP/VIP model). The reference angles of both joints were set to zero. The adopted stability criterion was that after an initial short transient (typically 5-10 seconds) and for a suitable observation time (at least 180 s) the orbits in the phase plane of both VIP and DIP/VIP models (*q*_*com*_ *vs*. ${\dot{q}}_{com}$) did not exceed the initial tilt. Table 3 shows the range of values of the control parameters, for both types of models, which support a stable sway motion.

**Table 3:** Range of stability of the intermittent controller.

	*P*/*mgh*	*D* [Nms]
SIP	0.5 – 0.9	0 – 430
DIP/VIP	0.3 – 0.9	0 – 470

The value of *P* is expressed as a fraction of the critical stiffness value for the SIP model (*m* denotes the total mass of the standing body and *h* the distance of the CoM from the ankle). The data reported in the table document that the intermittent control model is rather robust (for both models) because it allows a large range of variation. However, the DIP/VIP model is slightly better because it allows a larger range of variation. For most simulations reported, the following values were used:

$$\left\{ \begin{array}{l} P/(mgh)=0.6\\ D=70\;\mathrm{Nms} \end{array}\right.$$ (12)

The robustness of the intermittent feedback mechanism in complementing the intrinsic muscle stiffness is consistent with the study by van Soest et al. [28], which concluded that the combination of muscle properties and time-delayed spindle feedback is insufficient to obtain a system with reasonable local stability.

**Fig 3:**
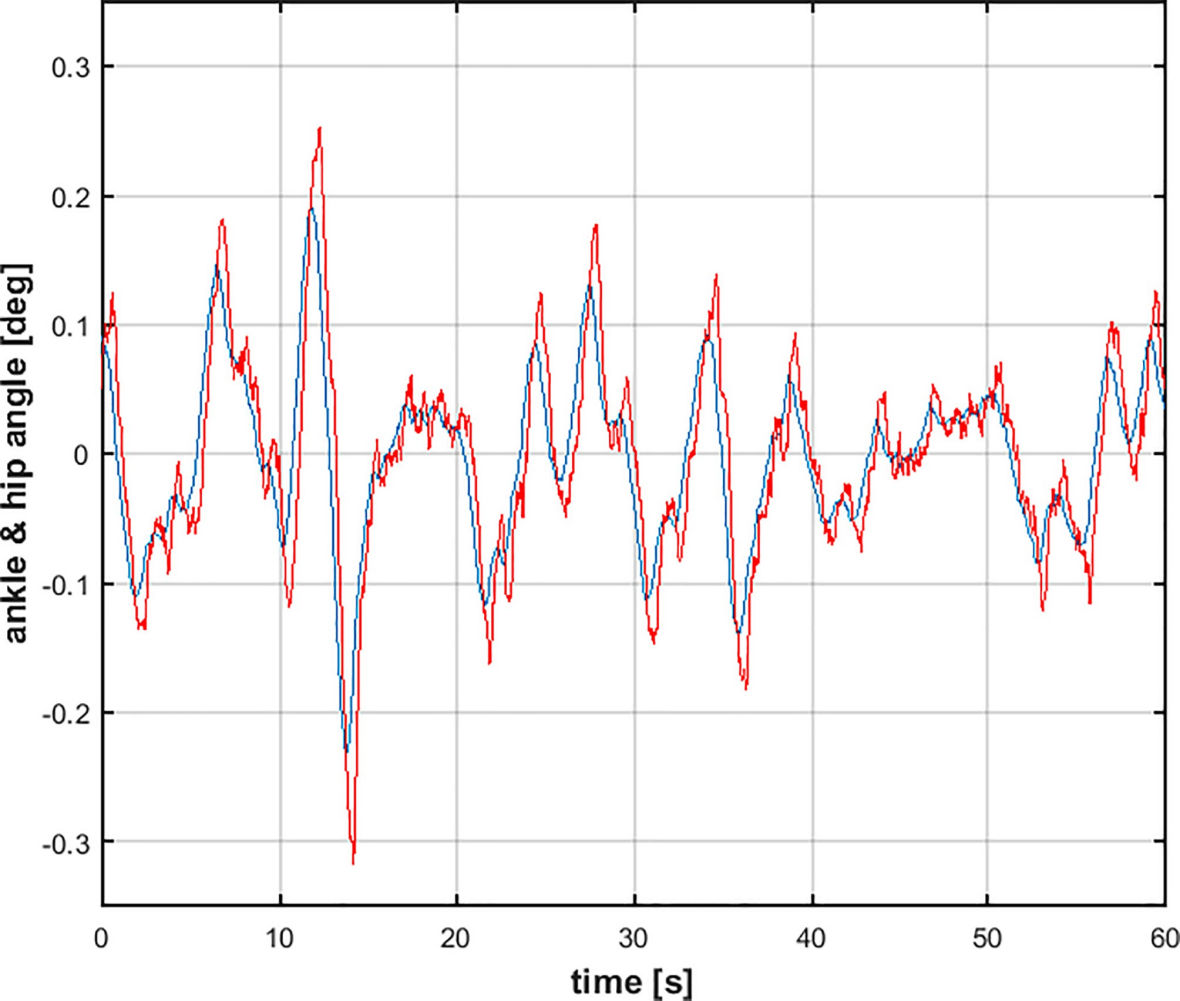
Sway movements generated by the simulation of the DIP/VIP hybrid model. Blue trace: ankle joint rotation; Red trace: hip joint rotation.

**Fig 4:**
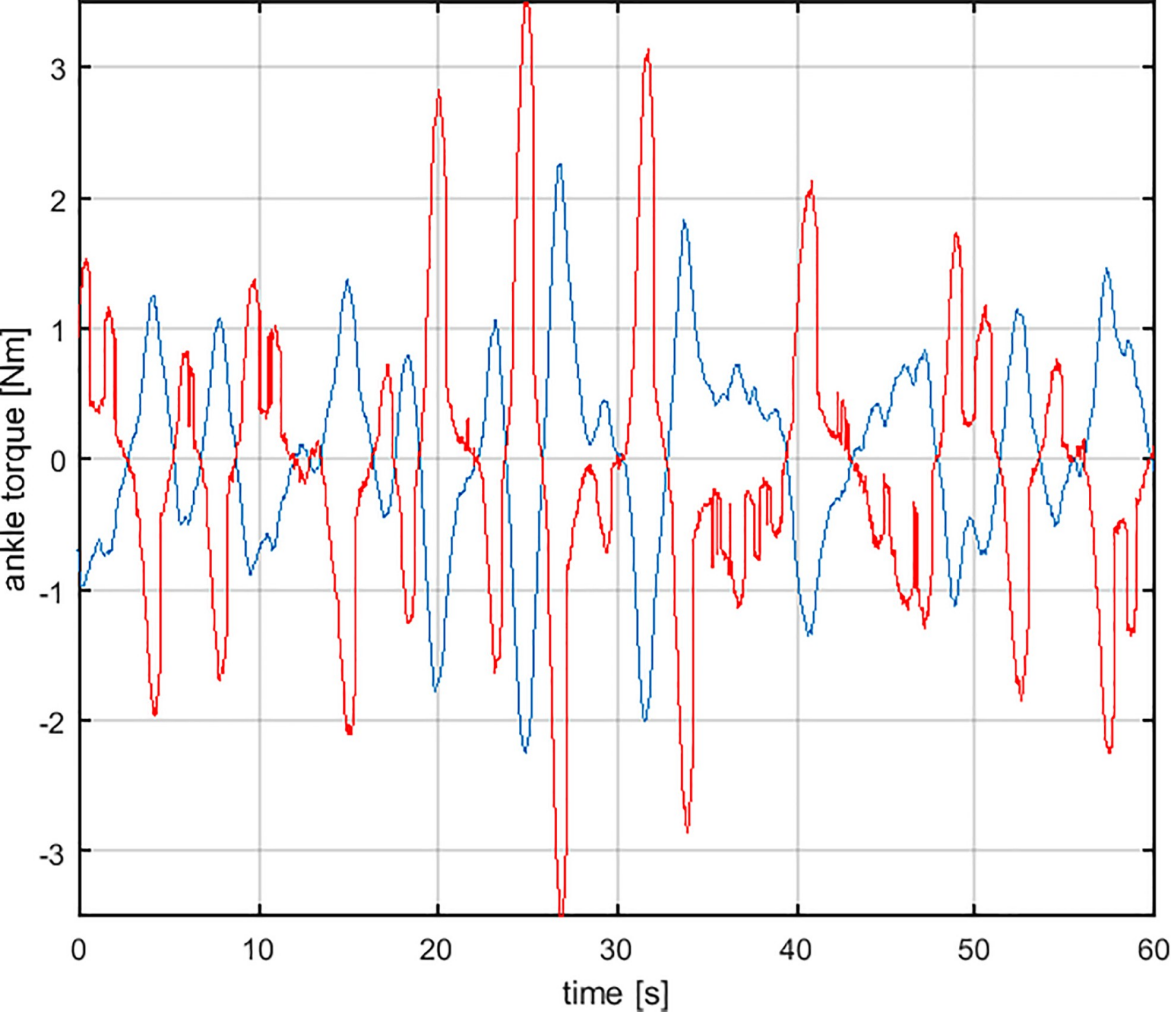
Stabilizing ankle torque generated by the simulation of the DIP/VIP hybrid control model. The DIP/VIP model (red trace) includes the stiffness component (passive, intrinsic, zero-delay feedback) and the intermittent control torque (active, delayed feedback). The counteracting ankle toppling torque due to gravity is displayed by the blue trace.

Fig 3 shows an example of sway movements (ankle and hip rotations) generated by the DIP/VIP hybrid model with the values above of the *PD* parameters and the other parameters listed in the model section. It appears that the oscillatory patterns of the two joints have a similar amplitude, emphasizing that the motion of the hip joint cannot be neglected, in agreement with what observed by many researchers [35,44,45,52–54]. Fig 4 shows the opposing torques acting on the ankle joint: the toppling torque due to gravity and the stabilizing control torque that combines the muscle/tendon stiffness effect and the crucial intermittent control action.

**Fig 5:**
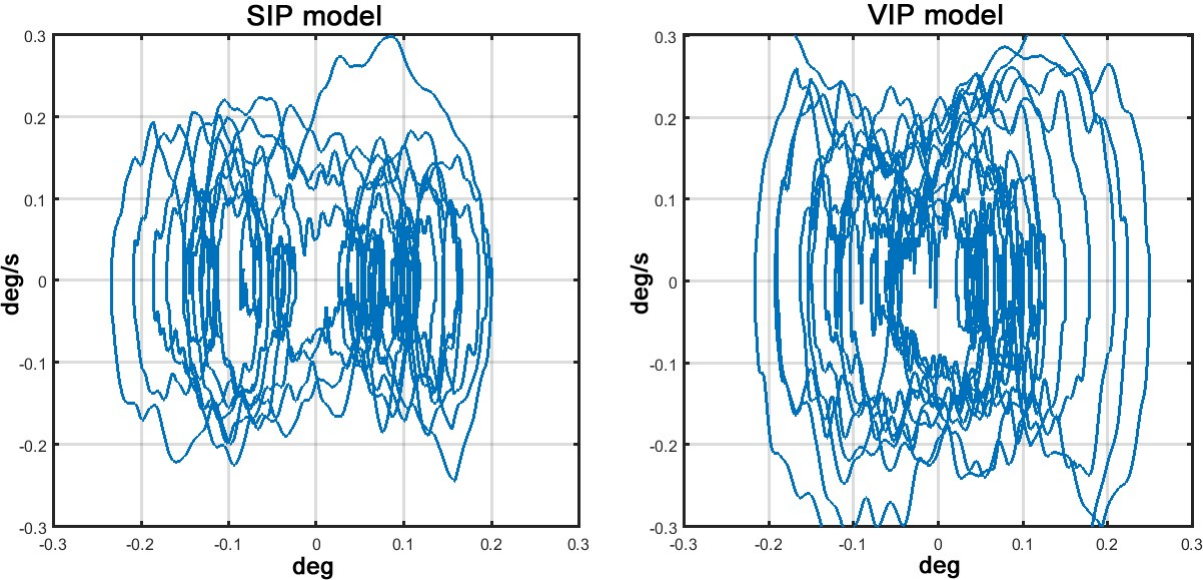
Sway orbits in the phase plane (*q*_*com*_ *vs*. ${\dot{q}}_{com}$). Left Panel: oscillations of the SIP model. Right panel: oscillations of the VIP part of the hybrid DIP/VIP model. Both orbits correspond to a 60 s sway. In order to better understand such similarity of VIP-SIP oscillations, we may consider the range of variation (RoV) of different variables during an observation window of 180 s, having defined as “range” the interval containing 90% of the samples stored during the observation time. Table 4 shows the RoVs for angular rotations, angular speeds and angular accelerations of the two models (DIP/VIP vs. SIP).

Although the previous analysis of the simulation results seems to emphasize the inadequacy of the SIP model to explain the massive contribution of the hip to overall sway movements, the comparison of the sway orbits in the phase plane of the SIP model and the VIP component of the DIP/VIP hybrid model (Fig 5) seems to tell a different story. The antiphase coordination of ankle and hip motion, first described by [44], tends indeed to minimize the acceleration of the global CoM [54] and thus the VIP oscillations of the DIP/VIP end up mimicking the oscillations of the old-fashioned single-DoF SIP model.

**Table 4:** Range of variation.

	DIP ankle	DIP hip	VIP	SIP
Angular rotation [deg]	± 0.11	± 0.13	± 0.13	± 0.20
Angular speed [deg/s]	± 0.17	± 0.34	± 0.20	± 0.19
Angular acceleration [deg/s^2^]	± 2.7	± 10.2	± 0.94	± 0.94

The observation of Table 4 reveals that in the case of the DIP model the RoV of the hip is systematically larger than the ankle: very small difference for the angular rotation, larger (twice) for angular speed, and very large (three times) for the angular acceleration. Such data are comparable with what was found empirically by observing the spontaneous sway of standing subjects [44,52]. Moreover, the theoretical study by [55] about multi-joint movement strategies based on biomechanical constraints indicates that the set of biomechanically feasible accelerations greatly favors a combination of ankle and hip movement in the ratio 1:3, in agreement with our simulations.

The RoV of the ankle motion in the SIP and VIP models is quite similar up to the first time derivative. In contrast, in the case of acceleration it is quite a different story: the angular acceleration of the ankle in the SIP model is much smaller than the acceleration of either joints of the DIP model and it is indeed almost coincident with the acceleration of the virtual VIP joint. Both our simulation results and the experimental results by [44] suggest that the main effect of the hip-ankle coordination is not to keep the CoM at a constant position, but rather to minimize its acceleration. In summary, such consistent ankle-hip coordination makes the behavior of the VIP component of the hybrid DIP/VIP model almost indistinguishable from the old fashioned SIP model.

**Fig 6:**
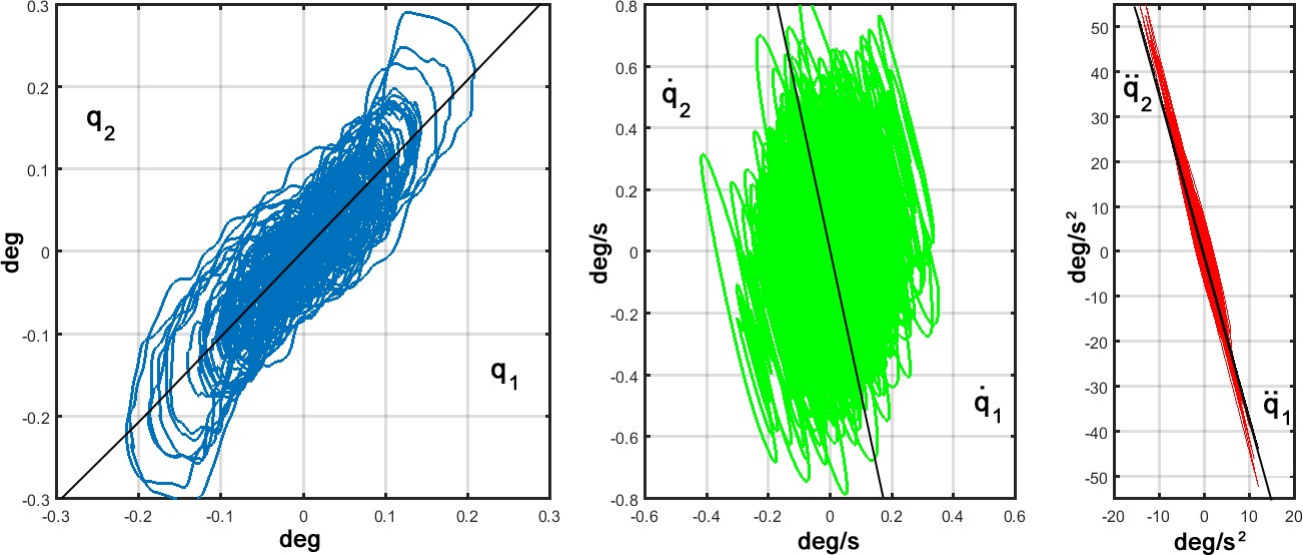
Coordination of Ankle (*q*_1_) and Hip (*q*_2_) oscillations from the simulation of the DIP/VIP hybrid model. The quantities showed are the angular rotations (left panel), the angular velocities (central panel) and the angular accelerations (right panel). The plotted traces correspond to a 60 s sway.

A third element of evaluation of the simulation results is more specifically related to the type of inter-joint coordination characteristic of the DIP model [53]. This relationship can be visualized by plotting ankle vs. hip motions separately for angular rotations, angular velocities, and angular accelerations, respectively (see Fig 6). The figure shows that angular rotations are positively correlated (the slope of the regression line is 1.02), whereas the trajectories of the angular velocity and angular acceleration exhibit a compensatory, anti-phase relationship (stronger for acceleration than for velocity): the slope of the regression line is -3.60 for velocity and -3.61 for acceleration. The results coming from our simulations are quite compatible with the experimental data of [44] for the experiments performed with closed eyes. In particular, the regression lines calculated from their experimental data have the following slopes: 1.32 (angular rotations), -3.40 (angular velocities), -3.12 (angular accelerations).

As a matter of fact, coexistence of both in-phase and anti-phase coordination patterns between the upper and lower body have been found in investigations which perturb upright stance as well in the analysis of quiet stance [45]: compare, for example, Fig 5 of [44], obtained from healthy subjects where knee and head-neck-trunk movements were restricted by suitable splints, with Fig 6 of our simulation. The coordination patterns of our simulations between states of the ankle and the hip fit well with the ones shown for representative experimental data in [44]. In particular, there is evidence of in-phase coordination for the low frequency components of hip and ankle rotations (up to 0.5 Hz) and anti-phase coordination for high frequency components (beyond 0.9 Hz). We verified to which extent this kind of correlation is compatible with the hybrid nature of the DIP/VIP model, without any specific arrangement in this direction in the formulation of the model. In order to carry out this analysis, the oscillatory patterns of the ankle and hip rotations generated by the model were low-pass and high-pass filtered by means of 4^th^ order Butterworth filters (with cutoff frequencies of 0.5 Hz and 0.9 Hz, respectively): remarkably we found a correlation coefficient equal to 0.7365 (in-phase coordination) for the low-frequency components and equal to -0.3996 (anti-phase coordination) for the high-frequency components, in agreement with the experimental data [44,50,53,56].

The hybrid DIP/VIP model is based on the combination of an explicit control mechanism (the intermittent controller) and an implicit mechanism based on the visco-elasticity of the hip muscle. As regards the latter mechanism, no direct measurement of hip stiffness is available, to our knowledge, and thus we do not have specific evidence about the fact that physiological values of this variable are indeed above the critical level for providing stiffness stabilization of the hip joint: ${K_{h}^{crit}=m}_{2}\;g\;r_{2}$ where *m*_2_ is the HAT-mass and *r*_2_ is the distance of the HAT-CoM from the hip joint. However, in the case of the hip joint the muscles are not connected to the upper body through very compliant tendons, as the Achilles tendon of the ankle joint. This fact, indeed, strongly limits the possibility of increasing the joint stiffness via co-contraction of antagonistic muscles in the ankle joint, as demonstrated by Loram et al [57]. Moreover, there are three significant issues in favor of the stiffness stabilization hypothesis of the hip joint:

1. the larger size of the hip muscles, in comparison with the ankle muscles, suggests a bigger value of the natural hip stiffness than the ankle stiffness, even without particular levels of co-contraction of antagonist hip muscles;
2. the critical value of the hip stiffness is much smaller than the ankle stiffness for purely biomechanical reasons (e.g. 165 Nm/rad vs. 823 Nm/rad with the anthropometric parameters used for the simulations of this study);
3. the coherence analysis of muscle activity during quiet stance [47] shows a lack of correlation between the oscillations of the trunk and the activations of the muscles, which exert direct control over it.

All these issues strongly support the physiological plausibility of a stiffness strategy for the hip in contrast with an active strategy for the ankle. The credibility of this hypothesis was also tested by evaluating the sensitivity of the hybrid control paradigm for a large range of variation of the hip stiffness above the critical level. First of all, we found that stability can be achieved for values of the hip stiffness at least 20% over the critical level. For values 20 times greater than the critical level the DIP/VIP model is practically coincident with the SIP model. As shown in the Table 5, when the ratio between the hip stiffness and the corresponding critical value is varied between 1.2 (the limit value for stability) and 20 (the value beyond which the DIP model behaves like a SIP) the slope of the regression line of the hip vs. ankle acceleration graph is moderately increased, while keeping the negative sign, i.e. maintaining the anti-phase relationship. Near the instability condition (hip stiffness only 20% higher than the critical value) the RoV of the three rotation angles (*q*_1_, *q*_2_, *q*_*com*_) is more than doubled, as expected for the influence of instability. For higher values of the hip stiffness, the RoV of *q*_1_ and *q*_*com*_ remain approximately constant, the former a little bit larger than the latter; in contrast, the RoV of the hip rotation *q*_2_ decreases as a consequence of the increase of hip stiffness, thus approaching a dynamical regime similar to the VIP model. It is quite surprising, on the other hand, that the slope of the regression line in the acceleration graph remains approximately constant, emphasizing the robustness of the hybrid control scheme for a very large variation of the hip stiffness. Moreover, the simulation results reported above provide an indirect estimate of the physiological level of hip stiffness adopted by standing subjects in absence of task specifications beyond the implicit one, namely “stand comfortably quiet”. The experimental data obtained in such condition [44,47,52–54] agree on the fact that the amplitude of the hip rotation is systematically larger than the ankle rotation. Since in our simulations this occurs if the hip stiffness is no greater than twice the critical stiffness, we feel confident to suggest that this kind of value may be a reasonable trade-off between stability and minimization of effort. Of course, this does not exclude that subjects may choose higher values of co-contraction for specific environmental conditions.

**Table 5:** Influence of the hip stiffness.

$\frac{Hip\;stiffness}{Critical\;stiffness}$	1.2	1.6	2	5	10	20
slope of regression line ${\ddot{q}}_{1}\;vs.{\ddot{q}}_{2}$	-3.4	-3.6	-3.6	-3.8	-3.9	-4.0
RoV *q*_*com*_ [deg]	± 0.295	± 0.144	± 0.126	± 0.114	± 0.129	± 0.120
RoV *q*_1_ [deg]	± 0.235	± 0.112	± 0.104	± 0.106	± 0.126	± 0.120
RoV *q*_2_ [deg]	± 0.590	± 0.195	± 0.126	± 0.036	± 0.019	± 0.009

## Discussion

It has been suggested [53] that the ankle-hip coordination during postural sway motion may be explained as an explicit attempt by the CNS to minimize the amplitude of the resultant angular acceleration of the CoM by applying a multi-joint optimal control paradigm. In particular, it is hypothesized that the ankle and hip torques are modulated in a temporally anti-phase manner to one another in each of the two joints in order to induce appropriate acceleration profiles. In this way, it is suggested that, by taking advantage of the inter-joint interaction, the CNS prevents the net torques from producing large amplitudes of the resultant angular accelerations. Implicit in this approach is that the old-fashioned SIP model is an over-simplification of a much more sophisticated postural control mechanism. However, we believe that this is not the only possible explanation of the observed coordinated patterns. The alternative explanation is that minimization of the CoM acceleration and the associated inter-joint coordination are not explicitly coded but are the implicit biomechanical consequences of the dynamical interaction between the actively stabilized lower body and the stiffness stabilized upper body, namely the hybrid VIP/DIP control model. The simulations performed in this study demonstrate that this simple model can explain inter-joint coordination without an explicit intervention of the CNS: thus the brain can address a single DoF stabilization problem quite similar to the one considered in the investigation of the old-fashioned SIP model. The difference is that the same intermittent control paradigm must be applied to a Virtual version of the SIP model, namely the VIP model that shares with the bi-axial DIP model the estimated position of the CoM. In addition to the empirical support to this explanation coming from the analysis of the simulation results related to the kinematics of coordinated sway patterns, an additional line of evidence is provided by the analysis of muscle activity during quiet stance carried out by [58]: they did not find any correlation between movements of the trunk and the activity of the muscles which exert direct control over it, whereas this correlation exists between ankle muscles and ankle oscillations. Thus the active intervention of the CNS seems to be limited to the ankle joint via the leg muscles (as implied by the SIP model) and the viscous elastic properties of the hip muscles determined by their tonic activity seem to be sufficient to stabilize the trunk, while inducing a characteristic hip-ankle coordination as a side effect of the overall dynamics.

There is also a paradoxical effect of the proposed extension of the SIP model to the VIP/DIP model. Our previous studies, which were focused on the intermittent control of the standing posture [36,37,40,41,59], were prompted by a criticism of the proposed stiffness control paradigm of balance in quiet standing [46], in the framework of a SIP model. The paradox consists of the fact that having accepted the biaxial nature of sway movements we offer support for a crucial role of stiffness control of balance. However the paradox is only apparent because we also suggest that the old-fashioned SIP model should be substituted by a more realistic DIP/VIP model with a hybrid control paradigm: active, intermittent control of the ankle joint and passive stiffness control of the hip joint. With such preliminary clarifications in mind we think that the old-fashioned SIP model is far from dead and indeed we agree with Gage et al. [60] who argue in favor of the “kinematic and kinetic validity of the inverted pendulum in quiet standing”. The SIP model is clearly a simplification of the more realistic DIP model but it is not an over-simplification because it captures the essential part of the DIP dynamics, provided that we accept a hybrid control paradigm.

On the other hand, we should clarify that the validity of the VIP model, associated with the hybrid control paradigm, is restricted to experimental conditions that allow the quick and sizeable production of ankle torque capable to transmit effective motion to the whole-body. This implies, in particular, that the support surface is rigid and of large area. Moreover, it seems natural to associate such requirements to the distinction between ankle and hip strategies for postural oscillations in the sagittal plane, that was formulated years ago by Nashner and McCollum [61]: they hypothesized that the ankle strategy stabilized the CoM by moving the whole body as a single-segment inverted pendulum by production of torque at the ankle; the hip strategy, in contrast, was supposed to move the body as a double-segment inverted pendulum with counter phase motion at the ankle and hip. They also suggested that the hip strategy should be observed in situations that limit the production of ankle torque, such as standing on a compliant surface, a behavior that was soon verified in reality [62]. However, we wish to point out that there is a strong difference between the ankle-hip coordination observed in such situation and the coordinated patterns considered in this study that assume a hard support surface: in the former case the range of motion of the CoP is strongly limited, whereas in the latter case it can exploit the whole length of the foot. Moreover, there is a reversal of the ratio between the amplitude of the oscillations of the CoP and CoM in the two oscillatory paradigms (it is greater than one in the ankle strategy and smaller than one in the hip strategy) as well as an inversion of the roles of the two variables (the CoP is the control variable and the CoM is the controlled variable in the ankle strategy, whereas the opposite characterizes the hip strategy). In order to complete this view about the great importance for the standing body of the contact with the ground, we should also take into account the recent study by Wright et al. [63] who clarified that rather than serving as a rigid base of support, the foot is compliant and sensitive to minute deformations, thus contributing to the stabilization of upright standing with the great sensitivity of a kind of incorporated force platform.

Summing up, the proposed modeling and control framework is directly applicable to the behavior of healthy subjects standing on a rigid surface, whose behavior is usually described in terms of the ankle strategy. On the other hand, one may speculate to which extent and in which sense this framework could be extended to interpret clinical and/or pathological conditions, as in the case of elderly people [64,65], people with low back pain [66], patients affected by somatosensory or vestibular loss [67], as well as general pathological conditions (multiple sclerosis, Parkinson’s disease, etc. [68,69]: clinical and/or pathological conditions that are frequently characterized by an enhanced presence of the hip strategy). As a matter of fact, the interaction of the two strategies and the mechanisms that may explain the shift from one strategy to the other were addressed in a previous theoretical work [70] that applied Intermittent control policies to the ankle and/or hip joints in a double inverted pendulum model. In particular, four types of model components were defined (off-off, on-off, off-on, on-on in relation with the ankle/hip pair of joints) where “on” means that the intermittent controller is active for the corresponding joint and “off” means that the active controller is switched off and the joint is partially stabilized only by passive resistance. The study showed that temporally coordinated active torque patterns, referred to as intermittent ankle, hip, and mixed strategies, can emerge by modulating the parameters of the active and passive model components. The modeling/control paradigm proposed in this paper may be considered a specific case of that theoretical study, related to the “on-off” model component, namely the organization of the postural stabilization system in which active (intermittent) control is limited to the ankle whereas the hip is stabilized in a passive manner by the viscous-elastic properties of the hip muscles. The innovation, with respect to the general approach of [70], is that the ankle controller takes into account the oscillation of the VIP, not of the leg per se. The underlying assumption is related to the fact that the brain has a reliable, although delayed, access to the state of the VIP as well as to the relative position of CoP and CoM over time. It is plausible that for a healthy subject standing on a rigid surface this kind of information is readily available, to a great extent via the sensing capabilities of the foot [63]. Modifications of the environmental conditions (increased compliance or decreased size of the support surface) as well as sensorimotor impairments may affect the reliability of the VIP bodily image thus forcing the brain to carry out a massive recruitment of body masses, as in the hip strategy, in order to equilibrate directly the CoM with respect to the CoP. In our opinion this is a promising research target, to be addressed with a combination of theoretical, modeling, and experimental studies.

## Supporting information

S1 Table Data from the simulation of the DIP/VIP hybrid model. In the Table are presented the angles and angular accelerations of Ankle (*q*_1_) and Hip (*q*_2_.).
(ZIP)
